# Marker-less real-time intra-operative camera and hand-eye calibration procedure for surgical augmented reality

**DOI:** 10.1049/htl.2019.0094

**Published:** 2019-11-12

**Authors:** Megha Kalia, Prateek Mathur, Nassir Navab, Septimiu E. Salcudean

**Affiliations:** 1 Robotics and Control Lab, Electrical and Computer Engineering, University of British Columbia, 2329 West Mall, Vancouver, BC V6T 1Z4, Canada; 2 Computer Aided Medical Procedures, Technical University of Munich, Boltzmannstraße 15, 85748 Garching bei Múnchen, Germany

**Keywords:** medical robotics, medical image processing, cameras, endoscopes, calibration, robot vision, surgery, phantoms, rendering (computer graphics), augmented reality, image registration, biomedical optical imaging, hand-eye calibration procedure, surgical augmented reality, pre-operative medical data, camera intrinsic matrix estimation, hand-eye transformation, camera calibration, endoscope, high visual error, virtual rendered tool tip, marker-less real-time intra-operative camera, augmented reality rendering, subsequent gradient descent steps, da Vinci robot, average target registration error, prostate phantom

## Abstract

Accurate medical Augmented Reality (AR) rendering requires two calibrations, a camera intrinsic matrix estimation and a hand-eye transformation. We present a unified, practical, marker-less, real-time system to estimate both these transformations during surgery. For camera calibration we perform calibrations at multiple distances from the endoscope, pre-operatively, to parametrize the camera intrinsic matrix as a function of distance from the endoscope. Then, we retrieve the camera parameters intra-operatively by estimating the distance of the surgical site from the endoscope in less than 1 s. Unlike in prior work, our method does not require the endoscope to be taken out of the patient; for the hand-eye calibration, as opposed to conventional methods that require the identification of a marker, we make use of a rendered tool-tip in 3D. As the surgeon moves the instrument and observes the offset between the actual and the rendered tool-tip, they can select points of high visual error and manually bring the instrument tip to match the virtual rendered tool tip. To evaluate the hand-eye calibration, 5 subjects carried out the hand-eye calibration procedure on a da Vinci robot. Average Target Registration Error of approximately 7mm was achieved with just three data points.

## 1 Introduction

Augmented reality (AR) and mixed reality (MR) are valuable technologies for medical applications. They can be used to render the medical data directly onto the patient's body for surgical planning and decision making. MR/AR improves the hand-eye coordination for the surgeon [1], but requires two calibration steps to take pre-operative medical data to the intra-operative camera/endoscope feed. These steps are: camera calibration, which determines the intrinsic camera calibration matrix, and a hand-eye calibration, that is the transformation between the endoscope coordinate frame to the camera coordinate frame. The hand-eye calibration requires camera calibration to locate points in the camera coordinate system.

One of the most widely used camera calibration methods in computer vision requires multiple images of a checkerboard pattern [2]. Such a method is not suitable for intra-operative use because inserting an external calibration pattern inside a patient's body has several drawbacks: it takes a long time, it requires deploying a larger pattern than the typical insertion hole, and requires bio-compatible and sterilisable calibration template materials. Therefore, in the past, multiple self-calibration methods have been proposed for intra-operative use, where the intrinsic camera calibration parameters can be estimated by using feature correspondences in the surgical scene [3, 4]. However, such methods cannot account for the change in lens distortion. Therefore, Pratt *et al.* [5] presented a calibration procedure where multiple pre-calibrations were performed pre-operatively and various intrinsic camera calibration parameters could be determined by using a single checkerboard image. The various camera parameters were modelled as a function of a single ‘focus position’. Unarguably much faster, such a calibration procedure still requires the endoscope to be taken out of the patient to capture the single calibration image. Additionally, the step needs an additional bed-side assistant or a resident doctor to perform the calibration. Moreover, during a surgery, surgeons move the endoscope and re-adjust the focus multiple times to get the best view of the surgical scene. In such a scenario it is a major disruption of surgical work-flow to take the endoscope out of the patient every time the surgeon re-adjusts the focus. In fact, the inability of new procedures to integrate seamlessly in the current existing surgical workflows is a major roadblock in translation of AR/MR surgical guidance systems to the operation theatre [6].

Therefore, with the aim of developing a real-time, intra-operative, marker-less camera calibration method, we adopted a similar approach as Pratt *et al.* (2014) with many key differences. We divided the calibration procedure into pre-operative and intra-operative steps. In the pre-operative step, similar to Pratt *et al.* (2014), we pre-calibrated the endoscope camera at multiple distances from the endoscope. The various intrinsic camera parameters and distortion coefficients were modelled as a function of the distance from the endoscope. However, unlike the previous work, we used a custom 3D printed camera calibration apparatus to eliminate errors and to ensure repeatability of the procedure. Moreover, we used a blur metric to isolate the most reliable focal plane from a multitude of possibilities. Furthermore, in intra-operative setting, we retrieved the entire camera calibration matrix without any external calibration object. We achieved this by determining the focal plane using the estimated distance between the endoscope and the surgical instruments working on the anatomy of interest. Unlike previous methods, our method can determine a reliable camera calibration matrix inside a patient's body without taking the endoscope out.

The second required transformation is the hand-eye transformation, which is sensitive to the change in focus. The existing state-of-the-art methods in robotics cannot be applied to surgical applications directly, as they require a calibration pattern [7, 8]. Therefore, finding a suitable hand-eye calibration method for minimally invasive surgical procedures is still a challenge and thus an active area of research [9–11]. Although finding the rigid body transformation between two coordinate systems requires just three points, additional points are usually required to find a reliable solution due to noise. This is a challenge, as selecting multiple points intra-operatively can take up significant surgical time. Therefore, many groups have proposed the hand-eye calibration methods by using a fewer number of points [9, 10] or by optimising the data collection process itself by minimising the predicted target registration error (TRE) [11]. Thompson *et al.* (2016) used an invariant point for hand-eye calibration. They placed a cross-hair near the operating surface and captured the images of the cross-hair by moving the laparoscope to fill the laparoscope's viewing volume. Then they estimated the hand-eye transformation using Levenberg–Marquardt least squares optimiser by locating the corresponding points in the laparoscope and the external tracking system. Shao *et al.* (2017) proposed a similar method where they used a invariant cross-hair as a marker. However, they progressively added the data points for the hand-eye estimation. The next step of the transformation estimation was initialised with the estimation of the previous step. It is important to note here that in both these cases, the calibration was performed *outside the patient's body*. Furthermore, before the hand-eye calibration, previous methods need a camera calibration step using a checkerboard calibration pattern. Thus, neither method can be performed inside a patient's body. Therefore, in this Letter, we present the first method for hand-eye calibration that does not require instrument tracking or intra-operative camera calibration. Unlike previously presented methods, ours is entirely compatible with surgical flow, and does not require additional targets nor removing the endoscope from the patients. Our method is iterative and gives a satisfactory visual accuracy with just three points.

Similar to camera calibration, the hand-eye procedure was divided into pre-operative and intra-operative stages. In the pre-operative stage, off-site, a hand-eye transformation was estimated by using a least square method [12]. Then, in the intra-operative step, a gradient descent algorithm was used to find the optimal rotation and translation. However, the data points were added sequentially and with each addition the algorithm was run again. In the first step the gradient descent was initialised with the hand-eye estimation from the pre-operative step. In subsequent steps, the initialisation was carried out with the estimation from the previous gradient descent steps. After each hand-eye estimate, the tool-tip was rendered on-screen to give immediate live visual assessment of the hand-eye accuracy. In the subsequent steps, only those points were collected where the visual error was high, thus minimising the number of required points.

Thus, to reassert, the major contribution of this work is to present a unified camera and hand-eye calibration method that neither requires an additional calibration pattern intra-operatively nor requires the endoscope to be taken out of the patient's body. We achieved this by collecting the rendered virtual tool positions that differed significantly from the actual visible surgical tool in the surgical scene. The method is real-time and estimated the hand-eye transformation with the minimal number of points. The camera intrinsic estimation can be performed inside a patient's body in <1 s. Unlike previous methods, our method does not require an additional bed side assistant for the calibration procedure. Thus, our method is practical for preserving the existing intra-operative surgical workflow. The evaluation was carried out by five subjects on a da Vinci surgical system. Furthermore, a surgeon was enrolled in a feasibility study where a prostate phantom was used to test the method, using a previously developed AR magnetic resonance-transrectal ultrasound (MR-TRUS) fusion system [13].

## 2 Materials and software

The system used for both the camera and the hand-eye calibration was a Windows 7 computer, with 3.60 GHz Intel(R) Core i7. OpenGL version 4.5 and C++ programming language were used for the renderings. The stereo video stream with graphics for the subject study was sent in through the surgical console using the SDI input.

**Fig. 1:**
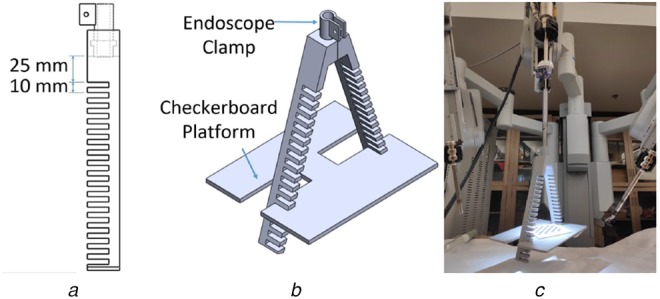
Apparatus used for preparation of multiple-depth camera calibration matrix *a* Apparatus used to mount the platform to perform camera calibration at various distances from the endoscope. The distance of the first level from the endoscope is 25 mm and the distance between the subsequent levels is 10 mm *b* CAD model of entire setup used for pre-operative camera calibration procedure *c* Camera calibration apparatus mounted on da Vinci endoscope

**Fig. 2:**
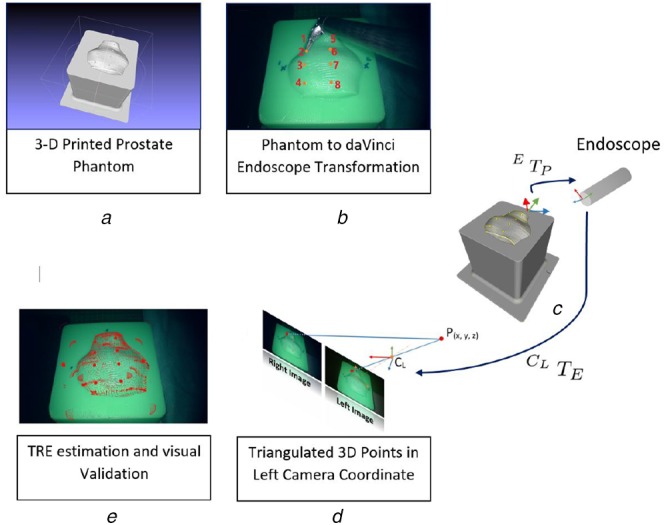
Hand-eye calibration evaluation procedure *a* 3D CAD model generated from a pre-operative MRI of a prostate of a real patient. Eight additional beads were added while designing the phantom for the purpose of the evaluation *b* Procedure of selecting eight beads using da Vinci surgical instrument on the surface of the phantom. The transformation between phantom mesh and the robot, $^R T_P$, was estimated by using these eight corresponding beads location *c*, *d* Transformation $^R T_P$, to bring the mesh points to robot coordinate system and hand-eye transformation, $^{C_L} T_E$, to further bring these points to the camera coordinate system *e* Visual validation by rendering the triangulated phantom mesh on the camera image using the estimated hand-eye transformation

For performing pre-operative camera calibration, the MATLAB stereo camera calibrator application was used. For evaluation, a 3D prostate phantom was designed using the pre-operative magnetic resonance image (MRI) of a patient's prostate (Figs. 1 and 2). The CAD model was designed in SolidWorks. For the calibration procedures and to estimate validation points in the endoscope coordinate frame, we used da Vinci Si surgical API [14] that gives the forward kinematics data from the robot. We used da Vinci Si system for user evaluation.

## 3 Methods

### 3.1 Camera calibration

#### 3.1.1 Camera calibration apparatus

In order to perform camera calibration, a custom 3D-printed fixture was designed to hold a checkerboard of known geometry in different poses relative to the da Vinci S endoscope. The fixture consists of an endoscope clamp and a platform that can be placed exactly perpendicular to the endoscope's axis at set distances. The clamp consists of a standard tube clamp design which allows the fixture to be secured to the camera without damage, as well as a stopper at the distal end to ensure consistent placement of the fixture. This stopper was carefully designed such that the stereoscopic cameras in the endoscope would not be occluded. The fixture has slots for the platform at known distances, starting at 25 mm with 10 mm increments up to 195 mm. This fixture design ensures the degrees of freedom of the checkerboard are limited to only rotation and translation in the plane perpendicular to the camera's axis.

#### 3.1.2 Preparation of multiple-depth camera calibration matrix

To prepare multiple camera calibrations for interpolation, we performed camera calibrations at various distances from the endoscope. To isolate the correct focal plane, i.e. the plane that is perpendicular to the optic axis, we used our custom made camera calibration apparatus that kept the calibration patterns perpendicular to the endoscope. To get a reliable camera calibration matrix it is essential to fill a significant proportion of the image with the checkerboard. However, the size of the checkerboard square decreases as the distance of the checkerboard from the endoscope increases. Therefore, we used checker-boards of different square sizes to carry out the calibrations at multiple depths. We empirically determined by considering the pixels covered by each square that the square size increases by ∼1 mm with each level of distance. Therefore, for the distances of 65, 95, 125, 155 and 185 mm, from the endoscope, we used checkerboards of square sizes 3, 5, 7, 9 and 11 mm, respectively. The range of distances was chosen by analysing previously recorded videos of radical prostatectomy procedures and estimating the distance of the endoscope from the surgical instruments. An interval of 30 mm was chosen so that there is no overlap between the subsequent calibrations when the pattern is moved in different orientations, to capture the images. Furthermore, to make the calibration readings consistent across various distances from the endoscope, checkerboards of the same thickness (1 mm) were used for various calibrations and for each calibration 25–30 images were captured.

To estimate the focal plane at each distance, we used the simple fact that the focused objects in real world are sharp and the blurred objects are out of focus. Therefore, before each calibration, the checkerboard pattern image in the endoscope was focused. To do so, we changed the focus of the endoscope by using the foot pedal in the da Vinci surgical console. However, determining the camera focus solely based on human blur perception cannot ensure a reliable repeatable focus value in subsequent steps of the camera calibration procedure. The reasons for this are two-fold. First, camera focus is a continuous value and, second, human blur perception and sensitivity varies across individuals [15, 16]. Therefore, to curb the variability in focus value we used a variance of the wavelet coefficients based blur measure [17]. It is a relative blur measure and when the value is maximum the image is considered to be in focus. To further make results of blur metric more consistent and background independent, we only considered the area covered by the checkerboard to calculate the blur measure. We placed the checkerboard in the centre of the platform and manually selected the region covered by the checkerboard. To reduce the computation time, the images were re-sized at this stage. The focus was changed continuously and the focal plane was selected based on its maximum blur value. This procedure took <10 s. Automation can further reduce the procedural time. Here, instead of manually selecting the checkerboard, the region covered by the checkerboard can be estimated automatically. The checkerboard region can be determined by an image threshold step, followed by morphological operations. The above method could be easily automated, starting by determining the edges of the checkerboard.

After selecting the focal plane we captured 25–30 images. Then, the calibration was performed using the method described in [2] using the MATLAB Stereo Camera Calibration toolbox. For each depth level, five calibrations were performed, or 25 calibrations in total (5 depth levels × 5 repetitions). To interpolate the parameters of the camera intrinsic matrix at the in-between depth levels, a fourth degree polynomial was fit to this data.

To evaluate the accuracy of the presented camera calibration procedure, we selected three focal planes, at distances of 85, 115 and 145 mm, and performed the camera calibration procedure using the method described in [2]. Three calibrations were performed for each distance. Thus, the validation dataset had, in total, nine calibrations. Then the results of curve fitting were evaluated on this validation dataset.

#### 3.1.3 Intra-operative camera calibration retrieval

In the pre-operative camera calibration step, different elements of intrinsic camera calibration matrix were parameterised using the distance from the endoscope. Given that the working distance from the endoscope is known during a surgery, we can retrieve the intrinsic camera calibration matrix. To estimate the working distance we make a reasonable assumption: where the surgical instruments are visible in the scene, that is the region of interest for the surgeon and that region should be focused. Thus, estimating the distance of the surgical instruments from the endoscope should give us a good estimation of the distance of focal plane. Therefore, in the intra-operative camera calibration step, we calculated the distance between the endoscope and the surgical instrument whose location is streamed in real-time from the da Vinci surgical API. The procedure took <1 s.

### 3.2 Hand-eye calibration

To estimate a rigid body transformation (i.e. rotation and translation), between two coordinate systems, we need just three corresponding points in both the coordinate frames. However, given noise, to get a reliable hand-eye transformation, more points are required using the currently available methods [9–11]. This is a challenge, as selecting multiple points during surgery can take crucial surgical time. Therefore, for seamless integration of any new calibration procedure in the existing surgical work-flow, it is important to find the transformation with a minimal number of points. With this goal, similar to the camera calibration procedure, we divided the hand-eye calibration procedure into pre-operative and intra-operative steps. Here we intend to estimate the hand-eye transformation in the intra-operative step using a minimal number of points, by ensuring a good estimation of the transformation from the pre-operative step. Due to physical constraints, the camera position does not change drastically with respect to the endoscope position. Thus, using a good initialisation from the pre-operative step, an iterative error minimisation algorithm such as gradient descent, would find an accurate hand-eye calibration with far fewer points compared to previous methods.

In a pre-operative stage, ∼60 corresponding points were located in the endoscope as well as the camera coordinate frame to find the transformation between the two. For locating the points in the camera coordinate frame, pixels corresponding to the surgical instrument tip, in the left and right image pair, were manually selected. Then, using stereo triangulation, the 3D world position of the surgical tool tip was estimated, with respect to the left camera frame. The corresponding position of the instrument tip in the endoscope coordinate frame was obtained using the forward kinematics data using the ISI surgical API of da Vinci surgical robot. Then a least square method was used to estimate the hand-eye transformation [12]. This step can be done before surgery.

**Fig. 3:**
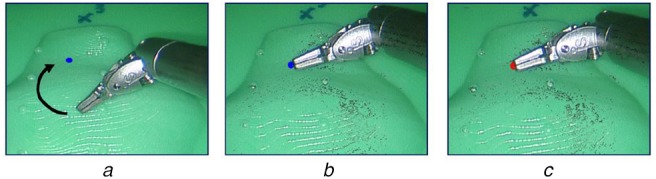
Procedure to collect data points, using virtually rendered dots, to find hand-eye transformation *a* Difference between the rendered surgical instrument-tip in blue and the actual surgical instrument tip *b* Procedure to take the actual surgical instrument tip to the incorrectly rendered tool tip, to collect a data point for the subsequent gradient descent step *c* Immediate visual feedback where the rendered dot turned red, when the user ‘touched’ the virtual point and the data point was collected

In a separate intra-operative step, an iterative gradient descent method was used. The method is similar to the one used in [18]. To calculate the sum of squared of residuals (SSR), Hersch *et al.* (2012) used any one point pair, $\lpar x_i\comma \; y_i\rpar $ at any given time, instead of using all the data points. Then the transformation was calculated to minimise the SSR using the gradient descent algorithm. However, our method differs from theirs by iteratively adding all the subsequent selected point pairs instead of just a single current point pair to calculate SSR. Thus, the procedure was started by locating just a single point pair in camera and the endoscope coordinate frame. The SSR was calculated for this point pair. To assess the accuracy of estimated hand-eye immediately, as feedback, the tool-tip was rendered on top of the camera image as shown in Fig. 3*a*. Then, in the subsequent step only those points were collected where the visual error was high between the estimated tool-tip and the actual tool-tip. To do this a ‘pause’ button was pressed to stop the movement of the blue rendered tool-tip. Then the user moved the surgical instrument to this poorly estimated tool position with the intention to ‘touch’ it. To give the feedback for ‘touching’ the rendered tool tip turned red, when the actual tool-tip occluded the rendered blue-dot in both right and left camera images. At this stage, the tool-tip position was recorded in camera and the robot coordinate frames. Then the new SSR was calculated by considering the current and the previous point. This was repeated until a hand-eye calibration, with satisfactory visual accuracy was obtained. This step, to collect data points using rendered virtual points, is crucial and enables our method to be implemented intra-operatively.

On average, just three points were sufficient for satisfactory results. The first step, took ∼30 gradient descent iterations while the subsequent steps took <6 iterations to reach the minima.

#### 3.2.1 Depth cues conflict resolution and visual feedback

As described in Section 3.2, data points were collected when the user successfully ‘touched’ the virtually rendered tool-tip for which the user was provided a feedback. Therefore, the accuracy of the current calibration to some extent depends on the 3D depth perception of the rendered point to facilitate the accurate reaching. Trivially rendered virtual point always render on top of the endoscope camera feed. The virtual point seems to be on top of the surgical instrument, in the camera feed, regardless of the position of the instrument in the real 3D world. This looks disturbing, especially in 3D, as stereo disparity and occlusion cues impart conflicting depth information. Therefore, to resolve occlusion, we created an occlusion mask for the instrument by thresholding the hue channel in hue-saturation-value colour space. Then we used this occlusion mask as alpha texture in OpenGL when rendering the surgical scene on top of virtual point. Thereafter, when the actual surgical tool occludes the virtual point, the occlusion can easily be detected simultaneously, in the left and the right image frames, to provide an instant visual feedback and the corresponding data point can be collected.

#### 3.2.2 Evaluation setup and validation data generation

Evaluation of the presented method is a challenge as there does not exist any gold standard method for intra-operative surgical applications. Furthermore, due to the interactive nature of the proposed method, inter-subject variability might affect the accuracy. Therefore, five subjects were asked to perform the calibration procedure, twice. Thus, the evaluation was carried out on ten repetitions of the calibration procedure.

For evaluation, a CAD model was generated from the pre-operative MRI of a patient. Additionally, in the phantom design eight visual marker beads were added. Then this model was 3D printed and brought to da Vinci endoscope coordinate system by manually locating these eight beads using da Vinci surgical instrument. Since the locations of the beads were known in both coordinate systems, a least square method was used to find the transformation $^R T_P$. These points were then brought to the robot endoscope coordinate frame according to the equation: $^E T_P = ^ET_R \times ^RT_P$. Here, $^E T_R$ is the transformation from the robot base to the robot endoscope, which was received from the da Vinci surgical API. Then in a separate step, we manually selected the pixels corresponding to the beads in the right and left image pairs. These pixels were used to estimate the 3D position of the beads using stereo triangulation with respect to the left camera. These eight points in the camera coordinate system and the da Vinci endoscope coordinate system were used to estimate the accuracy of hand-eye transformation using TRE and visual validation.

Finally, for the subject study, the phantom was placed on a table and the position of the endoscope was adjusted such that in the endoscope camera image, the phantom was oriented similarly to a real prostate in radical prostatectomy procedure. A previously recorded surgical video of the procedure was used as a reference.

### 3.3 Subjects and evaluation task

Since the evaluation can be affected by the level of dexterity in handling da Vinci surgical instruments, only the subjects who are comfortable using the da Vinci system participated in the evaluation. The subjects were graduate students who use da Vinci system as part of their research regularly.

The calibration procedure was carried out on a clinical da Vinci system. The subjects were seated at the surgeon's console to perform the procedure. In all of the reaching tasks, subjects used da Vinci surgical instruments by handling the master side manipulators as in a real minimally invasive (MIS) da Vinci robotic surgery.

Three crosses were marked and numbered on the phantom. Subjects were asked to reach the marked points in numerical order. Throughout the task, the tracked surgical tool using a pre-estimated hand-eye calibration was rendered as a blue dot. After reaching one of the marked crosses the tool tip tracking was paused, freezing the rendered tool tip in 3D space. Then, the subject was instructed to move the surgical instrument to the paused rendered blue dot. As soon as the subject ‘touched’ the blue virtual point the virtual point turned red. This gave an instantaneous feedback to the subject. This was achieved by the method described in the previous section. Then, using the iterative approach, the new hand-eye calibration was estimated and updated. With this updated hand-eye transformation the subject reached the second marked cross on the phantom and the procedure was repeated again. After collecting three points, the accuracy of the calibration could also be estimated by observing the tracked tool tip with the new hand-eye transformation. After each hand-eye estimation procedure, the accuracy was estimated by calculating the TRE on the previously described eight points and the results were visually validated by overlaying the phantom mesh on the endoscope camera image.

## 4 Results and evaluation

### 4.1 Camera calibration

The camera calibration re-projection error for each calibration while building the focus retrieval matrix was <0.8 pixels for the image size of $960 \times 540$.

The evaluation of curve fitting was performed on the validation dataset as described in Section 3.1.2. The root mean square error was calculated for each camera parameter. The results are shown in Table 1 for focus ($f_x\comma \; f_y$), principle point ($p_x\comma \; p_y$) and radial distortion coefficients, ${\rm ra}{\rm d}_1$ and ${\rm ra}{\rm d}_2$, for the right and the left stereo endoscopic camera. The focus and principle point are reported in pixel units.

**Table 1:** Results of the camera calibration evaluation

	Parameters	Left	Right
1	$f_x$	5.749	4.500
2	$f_x$	5.999	4.680
3	$p_x$	2.207	3.691
4	$p_y$	0.530	0.225
5	${\rm ra}{\rm d}_1$	0.013	0.010
6	${\rm ra}{\rm d}_2$	0.044	0.013

Throughout different camera calibrations, the translation and rotation between left and right stereo cameras were observed to be relatively constant. Mean translation, $\lpar T_x\comma \; T_y\comma \; T_z\rpar $ was found to be ($ - 5.61 \pm 0.050\, {\rm mm}$, $0.10 \pm 0.026\, {\rm mm}$, $ - 0.13 \pm 0.095\, {\rm mm}$). Furthermore, the maximum Frobenius norm of rotation matrix with identity matrix, for different calibrations came out to be 0.022.

### 4.2 Hand-eye calibration

In the pre-operative stage, the TRE for hand-eye estimation, using the least-square estimation, came out to be 4.70 mm. The intra-operative hand-eye accuracy was estimated on the validation dataset described in Section 3.2.2 through the subject study. The average TRE for each subject is shown in Table 2.

**Table 2:** TRE for hand-eye for each subject

1	2	3	4	5
3.08	7.48	13.30	5.44	5.73

The overall mean TRE was 7.01 mm with the SD of 3.85. The minimum TRE is 3.08 mm. For each subject the results were also visually analysed by overlaying the triangulated mesh of the prostate phantom on top of the prostate phantom endoscopic image (Fig. 3). Furthermore, the accuracy was also assessed visually by rendering the tool tip position on the camera image in real time. Table 3 shows a comparison of our method with relevant existing methods.

**Table 3:** Comparison of relevant hand-eye calibration methods

1	Thompson *et al.* (2016)	RMS projected error:
		optical tracking – 1.95 mm
		EM tracking – 0.85 mm
2	Shao *et al.* (2017)	Min. forward error – 1.32 mm (after 8 iterations)
		Min. backward error – 0.86 pixels
3	Chen *et al.* (2017)	Uniform sampling:
		TRE = 0.45 mm (*n* = 25)
		Guided calibration:
		TRE = 0.75 mm (*n* = 25)
4	Our method	Min. TRE – 3.08 mm (*n* = 3)

### 4.3 Feasibility of an AR TRUS-MR fusion system for radical prostatectomy procedure

To identify any unrealised issues with our methods, during a real surgery, we designed a feasibility study for da Vinci Si-assisted radical prostatectomy procedures using an AR MR-TRUS fusion system on a prostate phantom. The user was a surgeon, who routinely performs minimally invasive radical prostatectomy procedures, using da Vinci system. A Think Aloud Protocol was followed while the surgeon tried the AR system. The study also showed the practicality of our technique (Table 3).

**Fig. 4:**
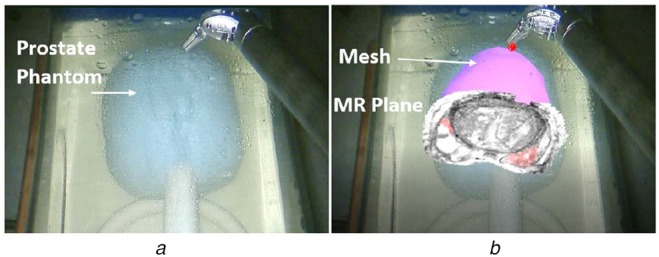
Feasibility study to test AR system for radical prostatectomy *a* Prostate phantom without AR view *b* Prostate phantom with the AR view of MR-TRUS fusion system. The prostate mesh was generated from the registered pre-operative MR to the intra-operative TRUS. The MR slice could be selected with the surgical instrument in real time to explore the MR volume intuitively. Tumours could be seen in red on the MR slice

First the registration of pre-operative MRI to TRUS of a prostate phantom was performed using the method described in [19] and then the USA was registered to the da Vinci coordinate system using the method described in [20]. Finally, our intra-operative camera and hand-eye calibrations were used to render the triangulated mesh generated from registered MR on top of the phantom. As shown in Fig. 4, the MR slice could be selected with the surgical tool-tip to better manoeuvre the 3D MR volume.

The surgeon's feedback can be summarised as follows: (i) the system was realistic and intuitive to use. The MR image looked like it was in the prostate and not just projected on top; (ii) it was easy to collect a few points for hand-eye calibration using the da Vinci surgical robotics system. It can easily be done intra-operatively and should not consume critical surgical time; (iii) as the rendered point was 2D, depth perception was a problem sometimes.

## 5 Discussion and conclusion

In this Letter, we presented a marker-less, intra-operative camera and the hand-eye calibration method. To the best of our knowledge, our proposed technique is the first unified camera and hand-eye calibration method that can be performed inside a patient's body without taking the endoscope out. Both the calibration procedures were divided into a pre-operative and an intra-operative step. For the camera calibration multiple calibrations were performed off-site and different calibration parameters were parametrised as a function of distance from the endoscope. Intra-operatively, the correct calibration parameters were retrieved by determining the distance of the surgical tool from the endoscope. Furthermore, for hand-eye calibration, in a pre-operative stage, the hand-eye transformation was estimated by using a least square estimation. In the intra-operative stage, we optimised the number of data points for the hand-eye estimation by sequentially adding only those points where the visual error was high, from a previous estimation step. A gradient descent algorithm was used where the first step was initialised using the transformation from the pre-operative stage. Using the estimated transformation, the surgical instrument-tip was rendered on screen to give immediate visual assessment of the transformation accuracy. In the subsequent steps, only those points were collected where this visual error was the highest. Thus, we obtained the optimal hand-eye transformation in as few as three points. After the first step, the subsequent gradient descent steps converged in <6 iterations.

The intra-operative camera calibration retrieval step took <1 s. This is crucial as camera calibration is a prerequisite for the hand-eye transformation. For the hand-eye calibration, as the result of the subject study with five subjects, the average TRE was 7.01 mm. However, satisfactory visual accuracy was achieved in each case. The result has SD of 3 mm. This relatively high variability can be due to the difference in depth perception among different subjects. Some subjects reported that the rendered virtual point, although looked 3D, was hard to assess for its depth accurately. In future work, this problem can easily be handled by rendering spheres instead of points and by using shading and shadow depth cues to enhance the perception of depth.

We also evaluated the intra-operative feasibility of our methods for minimally invasive radical prostatectomy. We presented an AR MR-TRUS fusion system rendered on the endoscopic camera feed, on a prostate phantom. The calibration procedures were carried out by a surgeon, who performs radical prostatectomy procedures routinely. The surgeon gave comments that the procedure was intuitive, easy to do and should be easy to perform during surgery. However, as many other subjects in the subject study, the surgeon found it slightly difficult to estimate the correct depth of the rendered dot. As mentioned already, the problem should be easy to solve by rendering spheres with shadow and other depth cues. In the future, the system will be evaluated on a real surgery.

## References

[1] Park M. Serefoglou S. Schmidt L.: ‘Hand-eye coordination using a video see-through augmented reality system’, Ergon. Open J., 2008, 1, (1), pp. 46–53 (doi: 10.2174/1875934300801010046)

[2] Zhang Z.: ‘A flexible new technique for camera calibration’, IEEE Trans. Pattern Anal. Mach. Intell., 2000, 22, pp. 1330–1334 (doi: 10.1109/34.888718)

[3] Dang T. Hoffmann C. Stiller C.: ‘Continuous stereo self-calibration by camera parameter tracking’, IEEE Trans. Image Process., 2009, 18, (7), pp. 1536–1550 (doi: 10.1109/TIP.2009.2017824) 19497819 10.1109/TIP.2009.2017824

[4] Stoyanov D. Darzi A. Yang G.Z.: ‘Laparoscope self-calibration for robotic assisted minimally invasive surgery’. Int. Conf. on Medical Image Computing and Computer-Assisted Intervention, Palm Springs, CA, USA, 2005, pp. 114–121 10.1007/11566489_15 16685950

[5] Pratt P. Bergeles C. Darzi A.: ‘Practical intraoperative stereo camera calibration’. Int. Conf. on Medical Image Computing and Computer-Assisted Intervention, Boston, MA, USA, 2014, pp. 667–675 10.1007/978-3-319-10470-6_83 25485437

[6] Linte C.A. Davenport K.P. Cleary K.: ‘On mixed reality environments for minimally invasive therapy guidance: systems architecture, successes and challenges in their implementation from laboratory to clinic’, Comput. Med. Imaging Graph., 2013, 37, (2), pp. 83–97 (doi: 10.1016/j.compmedimag.2012.12.002) 23632059 10.1016/j.compmedimag.2012.12.002 PMC3796657

[7] Tsai R.Y. Lenz R.K.: ‘A new technique for fully autonomous and efficient 3d robotics hand/eye calibration’, IEEE Trans. Robot. Autom., 1989, 5, (3), pp. 345–358 (doi: 10.1109/70.34770)

[8] Wengert C. Reeff M. Cattin P.C.: ‘Fully automatic endoscope calibration for intraoperative use’. Bildverarbeitung für die Medizin 2006, Hamburg, Germany, 2006, pp. 419–423

[9] Thompson S. Stoyanov D. Schneider C.: ‘Hand–eye calibration for rigid laparoscopes using an invariant point’, Int. J. Comput. Assist. Radiol. Surg., 2016, 11, (6), pp. 1071–1080 (doi: 10.1007/s11548-016-1364-9) 26995597 10.1007/s11548-016-1364-9 PMC4893361

[10] Shao J. Luo H. Xiao D.: ‘Progressive hand-eye calibration for laparoscopic surgery navigation’. Computer Assisted and Robotic Endoscopy and Clinical Image-Based Procedures, Québec City, QC, Canada, 2017, pp. 42–49

[11] Chen E.C. Morgan I. Jayarathne U.: ‘Hand–eye calibration using a target registration error model’, Healthc. Technol. Lett., 2017, 4, (5), pp. 157–162 (doi: 10.1049/htl.2017.0072) 29184657 10.1049/htl.2017.0072 PMC5683221

[12] Umeyama S.: ‘Least-squares estimation of transformation parameters between two point patterns’, IEEE Trans. Pattern Anal. Mach. Intell., 1991, 13, (4), pp. 376–380 (doi: 10.1109/34.88573)

[13] Samei G. Tsang K. Lobo J.: ‘Fused MRI-ultrasound augmented-reality guidance system for robot-assisted laparoscopic radical prostatectomy’. Hamlyn Symp. on Medical Robotics, London, UK, 2018

[14] Intuitive Surgical Inc. ISI API User Guide, 2014

[15] Maiello G. Walker L. Bex P.J.: ‘Blur perception throughout the visual field in myopia and emmetropia’, J. Vis., 2017, 17, (5), pp. 3–3 (doi: 10.1167/17.5.3) 10.1167/17.5.3 PMC5425112 28476060

[16] Green D.G. Powers M.K. Banks M.S.: ‘Depth of focus, eye size and visual acuity’, Vis. Res., 1980, 20, (10), pp. 827–835 (doi: 10.1016/0042-6989(80)90063-2) 7467137 10.1016/0042-6989(80)90063-2

[17] Yang G. Nelson B.J.: ‘Wavelet-based autofocusing and unsupervised segmentation of microscopic images’. Proc. 2003 IEEE/RSJ Int. Conf. on Intelligent Robots and Systems (IROS 2003) (Cat. No. 03CH37453), Las Vegas, USA, 2003, vol. 3, pp. 2143–2148

[18] Hersch M. Billard A. Bergmann S.: ‘Iterative estimation of rigid-body transformations’, J. Math. Imaging Vis., 2012, 43, (1), pp. 1–9 (doi: 10.1007/s10851-011-0279-x)

[19] Samei G. Goksel O. Lobo J.: ‘Real-time fem-based registration of 3-D to 2.5-D transrectal ultrasound images’, IEEE Trans. Med. Imaging, 2018, 37, (8), pp. 1877–1886 (doi: 10.1109/TMI.2018.2810778) 29994583 10.1109/TMI.2018.2810778

[20] Adebar T. Salcudean S. Mahdavi S.: ‘A robotic system for intra-operative trans-rectal ultrasound and ultrasound elastography in radical prostatectomy’. Int. Conf. on Information Processing in Computer-Assisted Interventions, Berlin, Germany, 2011, pp. 79–89

